# Pre-emptive Innovation Infrastructure for Medical Emergencies: Accelerating Healthcare Innovation in the Wake of a Global Pandemic

**DOI:** 10.3389/fdgth.2021.648520

**Published:** 2021-04-15

**Authors:** Khalil B. Ramadi, Shriya S. Srinivasan

**Affiliations:** ^1^Division of Engineering, New York University Abu Dhabi, Abu Dhabi, United Arab Emirates; ^2^Tandon School of Engineering, New York University, New York, NY, United States; ^3^Hacking Medicine, Massachusetts Institute of Technology, Cambridge, MA, United States; ^4^Department of Mechanical Engineering, Massachusetts Institute of Technology, Cambridge, MA, United States; ^5^Division of Gastroenterology, Hepatology and Endoscopy, Brigham and Women's Hospital and Harvard Medical School, Boston, MA, United States; ^6^Society of Fellows, Harvard University, Cambridge, MA, United States

**Keywords:** innovation infrastructure, translational medical research, health innovation system, pre-emptive innovation, hackathon

## Abstract

Healthcare innovation is impeded by high costs, the need for diverse skillsets, and complex regulatory processes. The COVID-19 pandemic exposed critical gaps in the current framework, especially those lying at the boundary between cutting-edge academic research and industry-scale manufacturing and production. While many resource-rich geographies were equipped with the required expertise to solve challenges posed by the pandemic, mechanisms to unite the appropriate institutions and scale up, fund, and mobilize solutions at a time-scale relevant to the emergency were lacking. We characterize the orthogonal spatial and temporal axes that dictate innovation. Improving on their limitations, we propose a “pre-emptive innovation infrastructure” incorporating in-house hospital innovation teams, consortia-based assembly of expertise, and novel funding mechanisms to combat future emergencies. By leveraging the strengths of academic, medical, government, and industrial institutions, this framework could improve ongoing innovation and supercharge the infrastructure for healthcare emergencies.

## Main Text

The COVID-19 pandemic has emphasized that ongoing innovation in healthcare is critical to meeting arising needs. Healthcare innovation, however, can be slow and costly (1) because of the complexity and risks involved. “Rapid” dissemination of health innovations are defined as those that disseminate in under 5 years (2). Drug development pipelines frequently take longer than a decade (3). While this pace pales in contrast to consumer technologies, it prevents spurious and poorly validated innovations from potentially harming patients. However, during emergency periods, such as a pandemic, the pace of innovation must be accelerated to decrease mortality and minimize economic impact.

Successful innovation in health care requires three distinct steps: (1) inception, (2) implementation/testing, and (3) dissemination (4). Efforts to spur the invention and inception of innovations, such as hackathons and workshops, have sprouted across industries and achieved significant success (5–8). Testing and implementation have been facilitated through the creation of incubators and accelerators that provide financial, logistical, and legal stability to early-stage companies. Diffusion, the practice of disseminating technology for widespread uptake (2), has been arguably the most underdeveloped step and presents a major bottleneck in the innovation process (2, 9–11), even fostering designation as an “art” (2). By definition, diffusion is a passive process, where adoption varies significantly on individual perception (12). While this represents the natural pathway in a free market, it makes healthcare innovation incredibly costly and challenging, ultimately at the cost of human life. How can we enable active, widespread adoption of innovations, in a more convective fashion? We have glimpsed what such a process could look like in recent approvals of various COVID-19 vaccines and devices, where innovations backed by multiple stakeholders were able to penetrate the market quickly, having rapid, and widespread impact.

**Figure 1:**
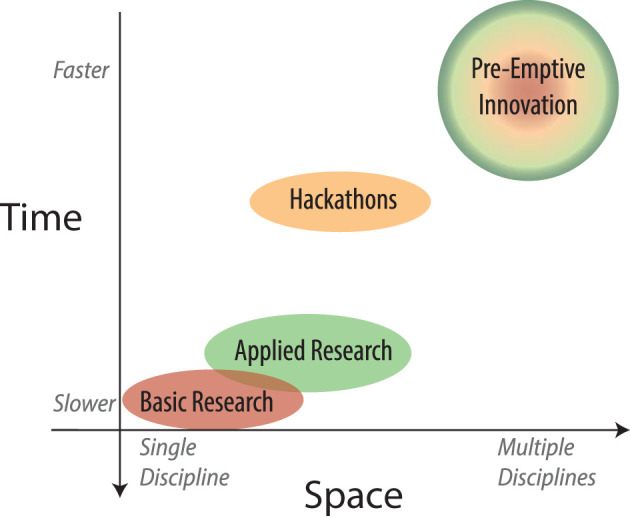
Comparison of various innovation processes along spatial and temporal axes.

Here we conceptualize a spatial-temporal framework (Figure 1) to describe the components and processes of innovation. By analyzing points of hand-off and inefficiencies in this framework, we propose a new mechanism to streamline innovation, specifically geared toward emergency preparedness, co-localizing critical components on both axes. Healthcare innovations can be products (e.g. drugs, medical devices), processes, or services. While the complexity and requirements for each differ, their progression can be mapped onto this framework.

## Spatial and Temporal Axes of Innovation

**Figure 2:**
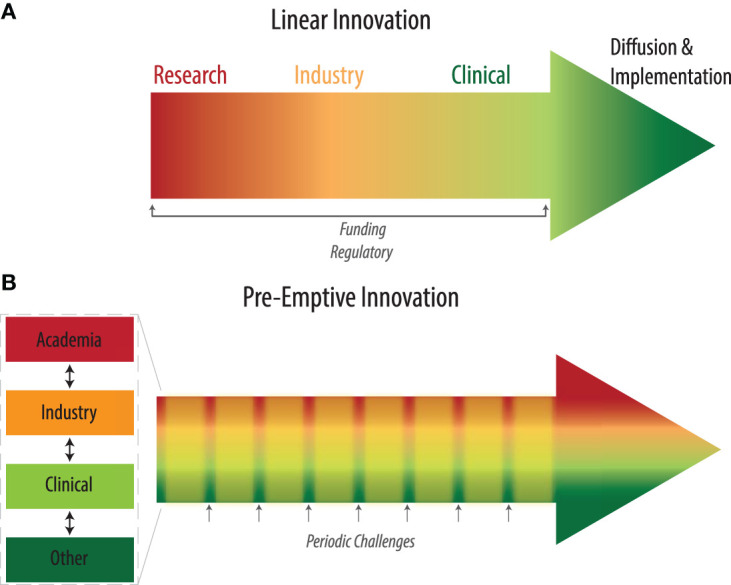
**(A)** Existing linear pipeline for health innovation. **(B)** Pre-emptive innovation pathway incorporating regular periodic challenges that engage all stakeholders throughout.

The healthcare innovation pathway can be examined from spatial and temporal perspectives. Prior temporal frameworks have highlighted the linear paths of innovation, from idea generation through adoption (4) while spatial frameworks have focused on the role of the adoption system, health system, and broader context (13). We seek to bridge these two frameworks to describe a holistic landscape (Figure 2A), incorporating (1) the distinct set of disciplines required for each step, (2) the ease with which different fields can interact, and (3) their respective incentive systems.

### Spatial Domains

#### Academia

Academic institutions have spearheaded multi-disciplinary and translational research through specific funding opportunities, creation of cross-departmental initiatives, and collaborative research centers. Basic science, engineering, and medical research are increasingly bridging fields that have been historically siloed. Multi-disciplinary academic and training programs play a central role in educating individuals to work at multi-disciplinary fields. An example is the Harvard-MIT Health Sciences and Technology (HST) program which integrates both engineering and medical training. At the interface of various disciplines, transformational devices and therapeutics are conceived, addressing challenges that conventionally-isolated disciplines were unable to tackle alone. Academic institutions provide depth in knowledge and skillsets, and generate substantially new ideas by students and faculty that come from a diversity of thought. However, innovation in academia is usually relatively slow. Academics are incentivized by publications, funding, patents, and overall impact. Research grants typically do not provide the funding required to translate and deploy innovations, although certain federal grant programs [e.g. National Institutes of Health (NIH)] have begun to offer this level of support.

#### Hospitals

Hospitals, and academic medical centers (AMCs) in particular, have incorporated a number of initiatives to enhance the integration of new health innovations (14–16). AMCs help determine the optimal method of integrating new innovations into existing healthcare pipelines and processes (17). AMCs have also developed innovation hubs that combine translational and clinical expertise to implement ideas. AMCs share incentives with traditional academic institutions, in addition to traditional hospital goals of improving patient care and patient experience while lowering overall costs.

#### Industry

Commercial entities offer expertise in scaling solutions beyond initial proof-of-concept testing. This expertise is vital for the dissemination of innovations. Industry can leverage deep expertise in these fields and significantly accelerate the pace of innovation. However, larger companies can struggle to switch focus between domains. How to manage such changes is the focus of extensive research (18, 19). Involvement of industry early in the innovation process can select for ideas and designs that can be marketed and sustainably manufactured at scale in the long-term. Innovations need to have a clear path to long-term financial sustainability to enable buy-in from for-profit corporations.

#### Payers

Payers play an important role motivating the development of new innovations, particularly those that improve long-term patient health while simultaneously reducing costs. Payers can be private insurance companies or government entities. Depending on the nature of the innovation, payers may not be directly responsible for covering costs, however the broad dissemination of innovations are likely to be enhanced if there exist insurance reimbursement codes, particularly for digital health technologies. Payer motivations and reimbursement structure largely determine the success of innovations.

#### Government

The role of government in health innovation can vary significantly depending on the national context. In countries with a centralized, public health system, government acts as the primary customer whose buy-in is crucial for innovation uptake. Where healthcare is privatized, governments traditionally only regulate approval of innovations. In both cases, government can often be a node that bridges different spatial players when specific needs arise. During the COVID-19 pandemic, e.g., the US government actively facilitated collaborations, provided funding and resources through Operation Warp Speed. Chinese and Russian governments actively aided the development of the COVID-19 vaccines.

### Temporal Domains

Most medical devices and therapies are conceived and preclinically validated in the academic realm over a period of years. Following the licensing or sale of intellectual property to a commercial entity, development of the product or service may take additional years before final testing via a clinical trial and regulatory approval. Large scale manufacturing and penetration into the market can further take a few years. Even for the most pressing of diseases, a timeline of 5 or more years is considered a reasonable time-to-market.

#### Innovation Challenges and Hackathons

Innovation challenges seek to expedite innovation by crowdsourcing solutions to problems (5–8, 20). Proposed problems can be open-ended (e.g. diabetes treatment) or have a specific focus of interest (e.g. multivitamin delivery in rural towns). Innovation challenges can occur over the course of a few days to a few months. Events generally incentivize the creation of teams that dedicate time and effort to developing ideas that may address existing problems in the sector. Hackathons are a specific type of innovation challenge where participants and teams are assembled and work together in a dedicated space for a prescribed length of time (21). This creates an environment where teams can enjoy protected time for focused, collaborative work. Innovation challenges also provide mentoring from experts across sectors, in order to answer questions teams may have and guide them as they refine and develop their ideas.

#### Incubators and Accelerators

Incubators and accelerators accept teams with ideas in various stages of development and guide them toward testing and implementation of their ideas, including pilot studies or clinical trials. These programs can provide funding, expertise, office space, and access to partners to facilitate testing. Incubators can exist as stand-alone entities or be housed within academic institutions. AMCs specifically have increasingly developed in-house incubator-like programs in an effort to cultivate internal innovation capacity (22, 23). Teams emerging from these programs often spin-out as commercial for-profit entities, raising funding from angel and venture capital sources, and successfully marketing and selling their products or services.

Each phase in the temporal domain is characterized by specific expertise. Basic science and engineering are required for invention and inception. Translational and clinical expertise are necessary to integrate and test innovations in specific clinical contexts. Business and manufacturing knowhow are crucial for scaling and sustainability. Temporally, the timeframes for each step can vary and depend, in part, on how rapidly different disciplines can be onboarded and convalesced into a multi-disciplinary team.

### Barriers to Diffusion of Health Innovations

Even the most successful health innovations face a steep battle in their diffusion and uptake. Previous studies have identified a number of barriers to health innovation diffusion including organizational structure, partnerships, lack of dedicated resources, and inadequate context (2, 9, 11, 12, 24–26). Proof of efficacy of an innovation does not guarantee its uptake. Rather, incentives for each active stakeholder need to be present to promote use. Rapidly-scaled innovations align these incentives (1) in an efficient manner.

A related challenge for organizations to incorporate innovations is to create an environment conducive to change management (10). Organizations need to create a climate for change and engage the whole organization in order to implement and sustain changes (18, 19). In healthcare, this challenge is amplified by the number of stakeholders involved.

The current paradigm of health innovations is much like a relay race, where each stakeholder takes the lead for one component of the translation process before handing it off to the next stakeholder. Each hand-off must contend with communication across disciplines that may each speak a different language. There also exists substantial inertia in each segment, given that innovation is often no one person's primary professional obligation, but rather a peripheral facet to their job (27).

In the face of a healthcare emergency such as the COVID-19 pandemic, the challenges enumerated above and the breadth of the spatial and temporal axes hinder innovation from being realized at a time-scale relevant to the situation.

### Pre-emptive Innovation Infrastructure for Medical Emergencies

We propose the concept of a pre-emptive innovation infrastructure for medical emergencies (PRIME) which proactively builds a sturdy cross-disciplinary ecosystem that is primed to spring to action in times of crisis. The PRIME would consist of teams in each geography that co-localize the various spatial domains and parallelize the temporal domains of innovation (Figure 2B). For each instance, state governments could identify key university, private-industry, and hospital labs in major academic cities to serve as the R&D hubs. Large companies with local manufacturing and/or prototyping spaces would be partnered with these labs for rapid prototyping, design, and development. Regulatory officials would be regularly involved to familiarize themselves to the community and articulate necessary testing requirements proactively. Within the team, technical, business, legal, clinical and regulatory leads would be appointed. As a pre-selected entity, the team would have the impetus to work together and move swiftly when called upon in times of crisis.

The PRIME is motivated by observations of how various players were able to ramp up innovation efforts during the COVID-19 pandemic. A shared sense of urgency and purpose during an emergency enabled businesses, institutions, and governments to deploy emergency protocols that accelerated the pace of work, regulatory and committee approvals, institutional review boards (IRBs) and other bureaucratic steps. It is also modeled after the multidisciplinary teams bridging academia, industry, and regulatory bodies assembled regularly by the Defense Advanced Projects Research Agency (DARPA).

The PRIME could be assigned predetermined pathways for testing, implementation, regulatory approval, scale-up, marketing, and funding to facilitate rapid implementation. Ownership, protection, and sharing of intellectual property would follow pre-templated models, based on the type of device or therapy, to prevent delays in innovation. During a medical emergency, directors of the PRIME would designate the major challenges, unlock government funding for R&D and facilitate advance purchase orders (APCs) to incentivize the manufacturing and deployment.

#### Challenge-Based Preparation

In non-crisis periods, PRIME teams would engage in regular innovation drills that challenge the networks to solve existing healthcare problems with transformational, as opposed to incremental innovations, similar to the Gates Foundation challenges. These could be funded through government grants and APCs, creating a market for qualifying solutions.

Similar to regular exercises carried out by the armed forces to ensure optimal preparation, these challenges would prepare all stakeholders for emergency scenarios. In addition to bolstering emergency preparedness, this infrastructure would establish the capacity for regular, constant cross-disciplinary collaboration and translation in healthcare, solving many of the large healthcare problems that we currently face. The PRIME allows professionals within each vertical to operate in such a way that meets their conventional incentive structures while simultaneously contributing to a broader, impact-driven schema.

#### A Climate of Change

PRIME tackles barriers identified by previous studies that impede health innovation (28, 29). Firstly, it fosters a culture of mutual understanding and co-creation across industries that may not be accustomed to regular collaboration, facilitating innovation flow (28, 29). This framework would also actively involve workers from each industry across levels and departments, creating a climate of change (30).

Each pathway of innovation espouses certain advantages and limitations. Ironically, a pathway that is too rigid can actively impede innovations that arise from nonconventional sources (22). Thus, we intentionally avoid prescribing a predetermined method of collaboration in the pre-emptive innovation infrastructure and encourage the creation of “slack,” whereby innovations from a variety of sources are nourished. Such slack can be created, e.g., by developing an open pathway whereby employees across industries can highlight problems and propose possible solutions. Employees could then be afforded some protected time from their primary responsibilities to pursue this idea, with appropriate guidance. This would also work toward changing organization structure to one of change for continuous quality improvement (31, 32).

The consolidated framework for implementation research (CFIR) has previously outlined various characteristics to facilitate implementation science. The weakest determinant was identified as the outer setting, external relationships across institutional and broader context in which innovations need to diffuse. PRIME would address this weakness directly (33).

Previous studies have identified that successful innovations require internal champions at every step of the innovation timeline to move them forward (34). A PRIME would take this one step further and obviate the need for individuals to move against the grain. Institutional frameworks would facilitate rather than impede innovative projects, while government and payer stakeholders would provide advocacy for emerging solutions. A sustainable health innovation ecosystem is a mechanism by which institutional entrepreneurship can be created across multiple players simultaneously (35). An established collaborative infrastructure would also facilitate data sharing and implementation, making diffusion and uptake of technologies less reliant on individuals and more intentionally integral to continuous healthcare improvement.

The race to develop devices and vaccines to address the COVID-19 pandemic has exemplified the potential of establishing sustained collaboration across stakeholders in healthcare for rapid innovation. The framework proposed here would be conducive to various types of innovations including digital innovations that have proven important in battling the ongoing COVID-19 pandemic (36). A PRIME would not only bolster emergency preparedness, but also catalyze ongoing innovation. Looking to the future, this infrastructure could also be applied to other impending crises including climate change, water scarcity, energy crises, and food security.

## Data Availability Statement

The original contributions presented in the study are included in the article/supplementary material, further inquiries can be directed to the corresponding author/s.

## Author Contributions

KR and SS jointly conceived the ideas presented here and wrote the manuscript. All authors contributed to the article and approved the submitted version.

## Conflict of Interest

The authors declare that the research was conducted in the absence of any commercial or financial relationships that could be construed as a potential conflict of interest.
